# First person – Naser Alshamsi

**DOI:** 10.1242/bio.062862

**Published:** 2026-09-28

## Abstract

First Person is a series of interviews with the first authors of a selection of papers published in Biology Open, helping researchers promote themselves alongside their papers. Naser Alshamsi is first author on ‘The neurotrophin DNT-2 via the Toll-2 receptor regulates neuronal survival and morphology during visual system development’, published in BiO. Naser conducted the research described in this article while a PhD student in Professor Alicia Hidalgo's lab at the University of Birmingham, UK. He is now a lecturer at the Mohammed Bin Rashid University of Medicine and Health Sciences, Dubai, United Arab Emirates, investigating the molecular mechanisms that regulate neuronal survival, development and connectivity, with a particular interest in evolutionarily conserved neurotrophic signalling pathways.

**Figure:**
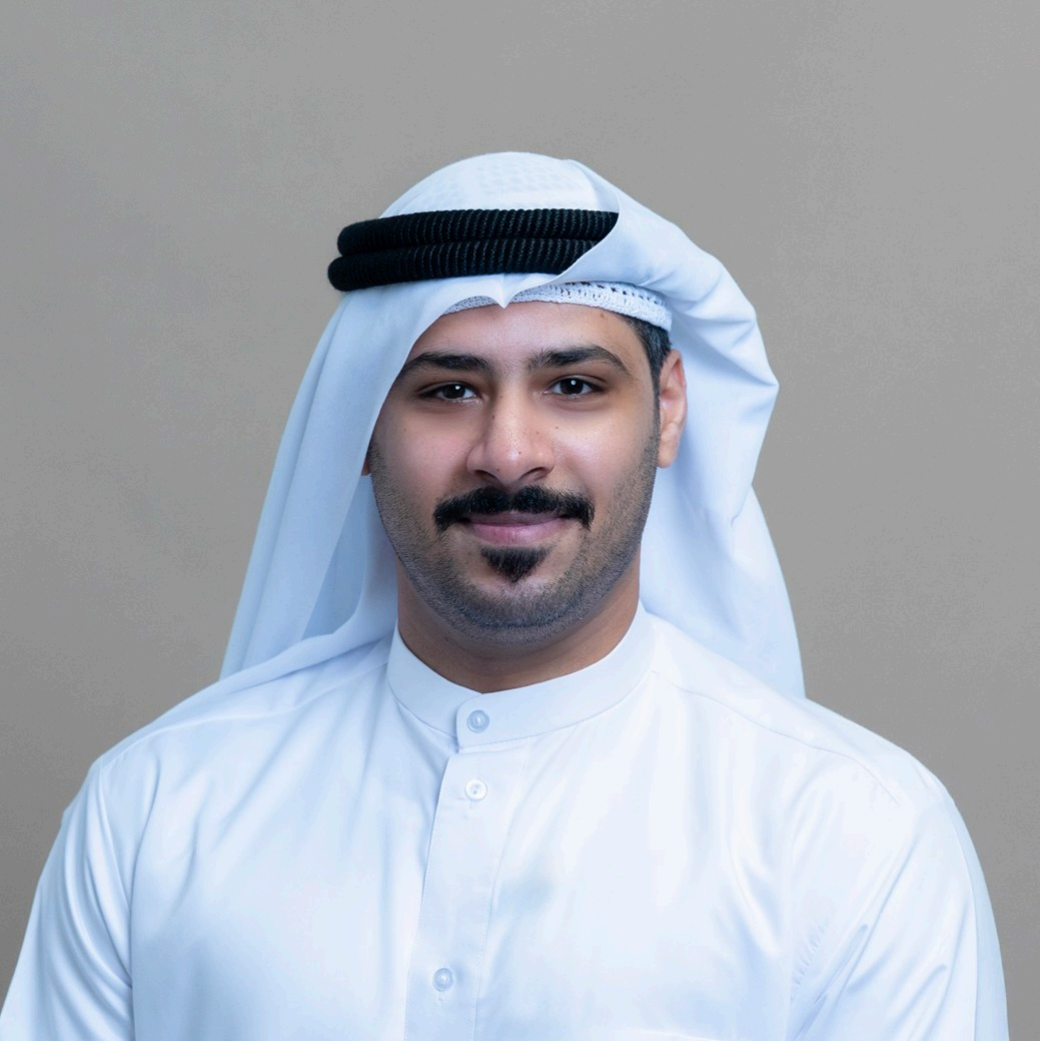
Naser Alshamsi

**Describe your scientific journey and your current research focus**

My scientific journey began during my Master's degree in England, where I joined the Hidalgo laboratory for my research project. The project gave me the opportunity to learn CRISPR gene-editing in *Drosophila*, which particularly appealed to me as a biologist interested in understanding and manipulating gene function. This experience sparked my interest in genetics and developmental biology and motivated me to continue in the same laboratory for my PhD. During my PhD, I shifted my focus towards neuroscience, investigating *Drosophila* neurotrophins and their roles in the development of the visual system. This work has shaped my broader research interest in understanding how genes and molecular signalling mechanisms regulate nervous system development and function.

**Who or what inspired you to become a scientist?**

During high school, I often felt that I was behind some of my peers in biology and chemistry. However, my chemistry teacher, Mr Jamal Attica, saw potential in me and encouraged my scientific curiosity and determination. He would often tell me that I would become a good scientist one day, and his confidence in me had a lasting impact on my decision to pursue science. Later, my PhD supervisor, Professor Alicia Hidalgo, played a similarly important role in my development as a researcher. During the early years of my PhD, I was still learning how to think independently, approach problems critically and design experiments on my own. Her trust in my abilities encouraged me to become more confident and independent as a scientist. Both experiences taught me how important mentorship and belief in a young scientist's potential can be.

**How would you explain the main finding of your paper?**

Our research aimed to understand how the brain knows how many neurons it needs to build neuronal circuits during development, to drive normal behaviour. In many contexts, the developing brain initially produces more neurons than it needs, and some are naturally removed while other neurons receive signals that help them survive and connect to other neurons. This process is controlled by neurotrophins – a type of growth factor – in humans and other vertebrates. Our lab had shown that neurotrophins are evolutionarily conserved in fruit flies, and that they regulate neuronal number in embryos and adult flies. We wanted to investigate whether this process similarly occurs in the developing visual system of the fruit fly, *Drosophila*. The adult visual system develops from larvae, and during metamorphosis there is a massive wave of cell death, during which key neurons need to be kept alive as the adult visual circuits are first established. Thus, the pupal visual system was an ideal context to test neurotrophin functions. In my project, we found that *Drosophila* neurotrophin −2 (abbreviated as DNT-2), produced by one type of neurons, works together with a receptor called Toll-2 in another type of neurons to support their survival in the visual system. Importantly, DNT-2 and Toll-2 also affect neuronal morphology, for example, the size and shape of dendrites. This raises the intriguing possibility that the control of neuronal survival by neurotrophins could be linked to the establishment of neuronal circuits also during the formation of the adult brain in insects. Our findings that the fundamental mechanism for building the nervous system through creating neurons in excess and then maintaining neuronal survival of the required neurons via neurotrophin ligands occurs recurrently through *Drosophila* development (i.e. embryos, pupa, adults), and thus has been conserved across evolution, from insects to humans.

**What are the potential implications of this finding for your field of research?**

Our findings provide further evidence that neurotrophism is an evolutionarily conserved mechanism that repeatedly regulates neuronal survival during nervous system development. We also identify DNT-2 as a candidate ligand for Toll-2, supporting emerging roles for Toll receptors in brain development beyond their well-known functions in immunity. Furthermore, our results suggest that different combinations of Toll receptors may give developing neurons distinct identities, helping them respond appropriately to neurotrophic signals and form robust neural circuits. These findings strengthen *Drosophila* as a model for understanding fundamental mechanisms of brain development and raise the intriguing question of whether similar interactions between neurotrophic factors and Toll receptors occur in mammals. Ultimately, this could provide broader insights into how the brain establishes and maintains correctly connected neural circuits and how these processes may be altered in disease.

**Figure:**
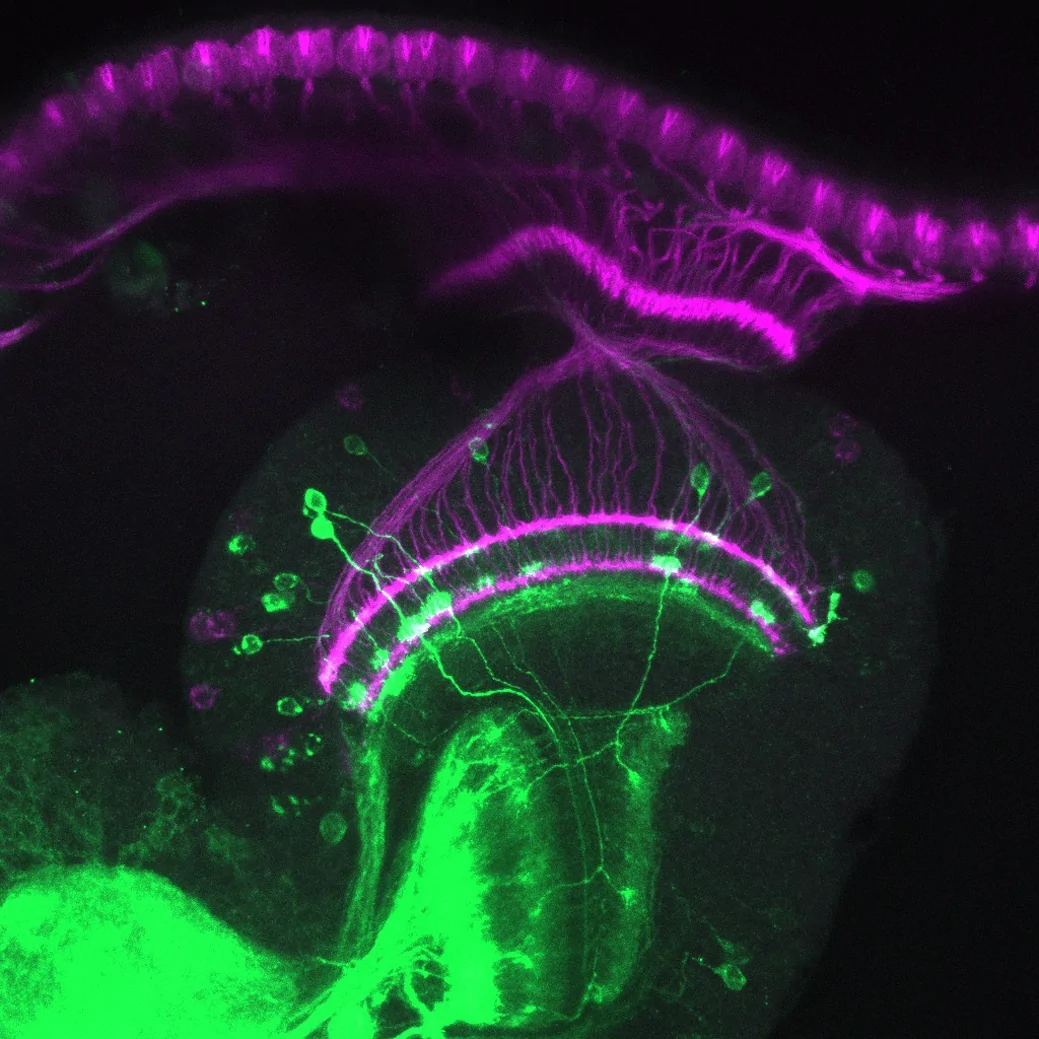
Confocal microscopy image of the developing *Drosophila* visual system, showing the intricate organization of neurons that form the visual circuit.

**Which part of this research project was the most rewarding?**

One of the most memorable moments of my PhD was the first time I saw neurons under a confocal microscope. Being able to visualise individual cells that form the complex networks underlying our actions and thoughts felt almost magical and strengthened my fascination with neuroscience. I also found attending conferences particularly rewarding. At several points during my PhD, we encountered questions that were difficult to address with the tools and expertise available to us. Meeting researchers at conferences who generously shared their expertise, particularly on the *Drosophila* visual system, as well as experimental tools and resources, helped us overcome some of these challenges. These experiences showed me how collaboration and the exchange of ideas can move science forward.

**What do you enjoy most about being an early-career researcher?**

What I enjoy most about being an early-career researcher is the opportunity to continuously learn and expand beyond my previous area of expertise. After working extensively with *Drosophila* during my PhD, I have begun exploring mammalian research and learning new techniques using mammalian and neuronal cell lines. This has allowed me to develop new approaches to studying gene function while building on the questions that first interested me during my PhD. I find it particularly exciting that a career in research provides the freedom to keep learning, explore new model systems and continually develop as a scientist.

**What piece of advice would you give to the next generation of researchers?**

My advice to the next generation of researchers is to embrace failure as part of the scientific process. During my PhD, I saw how failed experiments could be discouraging and sometimes make students question their abilities. I learned to view failure differently. When an experiment did not work or produced an unexpected result, I often felt a sense of excitement because it gave me an opportunity to dig deeper, ask new questions and search for an explanation. Some of the most interesting parts of research begin when things do not go as expected. I would encourage young researchers not to fear failure, but to approach it with curiosity, patience and the determination to understand why it happened.

**What's next for you?**

Since completing my PhD, I have returned home to the UAE and joined Mohammed Bin Rashid University of Medicine and Health Sciences (MBRU) in Dubai as a Senior Lecturer. I am now establishing my own research direction, moving into mammalian systems while continuing to explore questions related to those that fascinated me during my PhD. In particular, I am interested in investigating whether similar mechanisms involving Toll-like receptors operate in mammalian cells and how they contribute to nervous system function. Alongside developing my research, I have also begun teaching and mentoring medical, Master's and PhD students. This new chapter allows me to combine the two aspects of academia that I value most: pursuing scientific questions and helping to train the next generation of researchers.
